# Size Matters: How Scaling Affects the Interaction between Grid and Border Cells

**DOI:** 10.3389/fncom.2017.00065

**Published:** 2017-07-18

**Authors:** Diogo Santos-Pata, Riccardo Zucca, Sock C. Low, Paul F. M. J. Verschure

**Affiliations:** ^1^SPECS, Universitat Pompeu Fabra Barcelona, Spain; ^2^Institució Catalana de Recerca i Estudis Avançats Barcelona, Spain; ^3^Institut de Bioenginyeria de Catalunya (IBEC) Barcelona, Spain

**Keywords:** grid cells, border cells, error minimization, path integration, navigation

## Abstract

Many hippocampal cell types are characterized by a progressive increase in scale along the dorsal-to-ventral axis, such as in the cases of head-direction, grid and place cells. Also located in the medial entorhinal cortex (MEC), border cells would be expected to benefit from such scale modulations. However, this phenomenon has not been experimentally observed. Grid cells in the MEC of mammals integrate velocity related signals to map the environment with characteristic hexagonal tessellation patterns. Due to the noisy nature of these input signals, path integration processes tend to accumulate errors as animals explore the environment, leading to a loss of grid-like activity. It has been suggested that border-to-grid cells' associations minimize the accumulated grid cells' error when rodents explore enclosures. Thus, the border-grid interaction for error minimization is a suitable scenario to study the effects of border cell scaling within the context of spatial representation. In this study, we computationally address the question of (i) border cells' scale from the perspective of their role in maintaining the regularity of grid cells' firing fields, as well as (ii) what are the underlying mechanisms of grid-border associations relative to the scales of both grid and border cells. Our results suggest that for optimal contribution to grid cells' error minimization, border cells should express smaller firing fields relative to those of the associated grid cells, which is consistent with the hypothesis of border cells functioning as spatial anchoring signals.

## 1. Introduction

Having optimal navigational strategies is key for survival in complex and ever-changing environments. For an animal to do so, it requires the encoding of its surroundings into reliable internal representations and the ability to recall spatial memories. The rodent hippocampus has been a popular subject of investigations aimed at revealing the neural circuitry engaged in such mnemonic and navigational processes, especially as it serves as a reasonable and economical comparison to the functions of the hippocampus in humans and other primates (see Squire, 1992; Burgess et al., 2002; Brown et al., 2016 for a review on the human hippocampus and the generalizability of rat hippocampal studies to primates and humans).

It is now well-established that rodent hippocampal place cells have their receptive fields tuned to specific spatial locations, allowing them to situate themselves in space (O'keefe and Nadel, 1978). This internal representation receives contributions from grid cells located in the late layers of the medial entorhinal cortex (MEC) that tile the explored environment with characteristic hexagonal firing fields and provides a sensory-independent spatial metric system (Hafting et al., 2005).

The input-output transformation from grid to place cells has been addressed by different computational models. One such example is de Almeida et al. (2009), where the authors proposed that place cell-like activity emerges when the activity of grid cells in an environment, with overlapping, distinct scales and orientations, is summed and combined with network competition mechanisms. The resultant metric mechanism is sufficient to encode the animal's current position in space.

The underlying mechanism for grid cell formation has been commonly suggested to be continuous attractor networks (CAN), which are based on recurrent connectivity among cells within a neural sheet creating clustered bumps of high activity in proximal cells (Guanella et al., 2007). CAN models are sensitive to synaptic modifications, which shift attractor points within the network, effectively making bumps of high activity move along the neural sheet. To generate an internal spatial metric system representing the agent's location, CAN models of grid cells integrate the current velocity of the agent with the lateral synaptic connectivity. Indeed, velocity-related components have been experimentally recorded in the rodent MEC (see Giocomo et al., 2014; Kropff et al., 2015). In this way, movements of the neural activity bump in the CAN will resemble those of the agent in the world. Given that grid cells cover the entire environment with repeated firing fields, configuring the topology of the network into a toroidal architecture allows for periodic firing at multiple spatial locations and, thus, the generation of the characteristic hexagonal tessellation pattern (Guanella et al., 2007).

Evidence of low-dimensional continuous attractor dynamics underlying grid cells' formation has been observed during extracellular recordings from grid cells in rodents performing a navigational task (Yoon et al., 2013). As spike activity between pairs of cells tends to maintain a constant spatial relationship, from stronger synaptic weights between closer cells to weaker weights between distant cells independently of the animal's experience in a given environment, attractor models seem to be a plausible approximation of grid cells' activity. Further supporting evidence comes from the *in-vitro* observation of slow ramps, a typical signature of attractor dynamics, conducting both cellular and network behavior of grid cells in the rodent MEC (Domnisoru et al., 2013).

### 1.1. Error accumulation and alleviation

A key aspect of the attractor-based models of grid cells is their dependency on velocity signals as the main drivers of the activity bumps. However, the physical properties of sensory acquisition processes and neural instability inevitably lead to an accumulation of errors over time (Burak and Fiete, 2009). Error accumulation has been of particular interest in the field of robotics, and the common solutions proposed to minimize it are generally sensor fusion (Julier and Uhlmann, 1997; Kam et al., 1997; Lynen et al., 2013). In rodents' grid cells, such accumulation of errors has also been reported (Hardcastle et al., 2015). When traversing an environment, grid cells accumulate a drift in their firing fields. When the animal approaches the boundaries of the environment, this drift is reset, suggesting that border cells may play a role in grid cells' error minimization. In the same study, a computational mechanism was proposed in which border cells' Hebbian activity, paired with grid cells' activity, minimizes errors based on path integration when the agent is closer to the environmental boundaries. In other words, environmental boundaries provide spatial references to offset errors accumulated during spatial exploration.

The idea that spatially-tuned hippocampal cells enable a reset of accumulated errors in grid cells was first addressed by Guanella et al. (2007). It was predicted that feedback projections from the hippocampus proper to grid cells would anchor grid cells' activity to specific spatial locations, thereby resetting the accumulated error to the ground truth. Subsequently, experimental evidence for this was found *in-vivo*, with disrupted hippocampal-MEC projections resulting in a loss of the stereotypical hexagonal pattern of grid cells (Bonnevie et al., 2013). Hippocampal-MEC projections do not directly target grid cells (Naber et al., 2001). This thus raises the possibility that such a disruption indirectly affects border cells' stability, which, in turn, does not properly contribute to grid cells' error minimization leading to grid cells losing their characteristic hexagonal pattern.

### 1.2. Scaling in spatially tuned cells

Grid cells show a progressive increase in scale along the MEC's dorsal-to-ventral axis (Brun et al., 2008; Kjelstrup et al., 2008). Neurons found in the dorsalmost regions exhibit small-scale tessellation, with receptive fields spanning ~0.3 m. Grid cells located more ventrally tile the environment at a larger scale, and therefore at a lower resolution, with distances between firing fields spreading up to 3 m (Brun et al., 2008). From a computational perspective, multiple scales allow input-output transformations to tune place cells to specific locations in the environment (de Almeida et al., 2009); in other words, such a scaling property allows rodents to build their own internal representation of space in non-sensory environments (Markus et al., 1994; Moser et al., 2008). From a representational perspective, small-scale grid cells would encode the environment at a higher resolution, allowing for precise decoding of nearby spatial locations. On the other hand, large-scale grid cells would permit the linking of distant locations with less computational effort.

The progressive scaling property is not unique to grid cells; it has also been observed in other cells in the hippocampal formation. For example, head direction cells found in dorsalmost regions of the MEC are tuned to specific head orientations, while those located ventrally are most responsive to broader, but fixed, global orientations (Giocomo et al., 2014). In addition, place cells found in the hippocampus proper distribute their activity specificity along the hippocampal dorsal-to-ventral axis (Giocomo et al., 2007). Again, firing fields of neurons located at dorsalmost regions represent small and highly tuned locations, while ventralmost cells display broader and less location-specific firing activity. Hence, the progressive scaling phenomenon seems to be a general property of the rodent hippocampal representational system.

Earlier implicated in the reduction or elimination of error accumulation in grid cells during active exploration of enclosures (Hardcastle et al., 2015), border cells found across MEC layers likely interact with grid cells (Solstad et al., 2008). Considering how they are functionally related to neurons which display the aforementioned scaling property, border cells could be similarly scaled across the dorsal-to-ventral axis. As border cells encode environment boundaries, and environment boundaries contribute to grid cells' error minimization, a pertinent question would be: would the grid cells' error minimization mechanism benefit from scale-modulated border cells?

The more the interaction between grid and border cells enables grid cells to maintain accurate firing fields over time, the more beneficial it is in the context of error minimization. If we consider the firing field of a border cell as an anchoring signal to specific locations in space, border cells with small firing fields would precisely encode specific locations. On the contrary, border cells with large firing fields would be less accurate in signaling a given environmental position. Thus, we should expect that grid cells would benefit more from border cells expressing smaller scales, with large-scale border cells being detrimental to small-scale grid cells as the border signals would cover larger areas than the grid fields themselves. To wit, it is expected that the scale of grid cells should be comparable with that of the border cells which they interact directly with.

To examine how a progressive increase in border cells' scale along the dorsal-to-ventral axis of the MEC would affect grid cells' error minimization, we built on a previously presented computational model of grid cells accounting for this scaling property combined with an activity signal mechanism mimicking the role of border cells involved in error minimization of path-integrating grid cells (see Guanella et al., 2007; Pata et al., 2014; Maffei et al., 2015). By observing the effects of multiple border cells' scaling factors, we could test whether border cells with distinct scales would optimize error minimization of grid cells.

## 2. Materials and methods

### 2.1. Computational models

#### 2.1.1. Grid cell model

Low-dimensional continuous attractor dynamics approximate the underlying organization of grid cell networks in the mammalian brain (Yoon et al., 2013). In this study, we use a previously described model of grid cells formation based on attractor dynamics with synaptic connectivity following a toroidal topology (Guanella et al., 2007; Pata et al., 2014; Maffei et al., 2015). The model comprises five subpopulations of grid cells, each with a specific grid scale, mimicking the physiological properties of progressive scale increases along the dorsal-to-ventral axis (Kjelstrup et al., 2008).

Each population is made of 400 rate-based cells recurrently connected, and at every simulation time step (*dt* = 1 ms) the velocity vector of a simulated agent is integrated onto the network's dynamics through the modification of grid to grid synaptic weights. The network is initialized with uniformly random activity between 0 and 1/*N* (where *N* is equal to the number of cells in each subpopulation). The activity of cell *i* at time *t* + 1, i.e., ${A^{\prime }}_{i}(t+1)$, before the integration of border cells' activity, is updated at every simulation cycle *t* through a linear transformation function *B*_*i*_(*t* + 1) of the form:

(1) $$\begin{array}{c} B_{i}(t+1)={A^{\prime }}_{i}(t)+\overset{N}{\underset{j=1}{\sum }}{A^{\prime }}_{j}(t)w_{ij} \end{array}$$

where, *w*_*ij*_ denotes the synaptic weight between cells *i* and *j*, with *i*, *j* ∈ {1, 2, …, *N*}. *N* is the number of neurons in the network, $A_{i}^{\prime }\left(t\right)$ is the activity of a given cell *i*, $\sum \left(A_{j}^{\prime }\left(t\right)\right)$ is the activity of cells connected to cell *i*. To guarantee the stability of the network activity and prevent it to grow exponentially, an average normalization mechanism is applied. Finally, $A_{i}^{\prime }\left(t+1\right)$ is defined by:

(2) $${A^{\prime }}_{i}(t+1)=B_{i}(t+1)+\tau \left(\frac{B_{i}(t+1)}{<B_{j}(t){>}_{j=1}^{N}}-B_{i}(t+1)\right)$$

where $<B_{j}\left(t\right){>}_{j=1}^{N}$ is the network's mean activity. To avoid negative activity values, the activity $A_{i}^{\prime }\left(t+1\right)$ is set to zero when $A_{i}^{\prime }\left(t+1\right)<0$. The parameter τ determines the stabilization strength of the network. The parameters of the model are summarized in Table 1.

**Table 1:** Parameters used in model.

**Parameter**	**Value**	**Units**
N (per module)	20 × 20	Cells
M	5	Modules
Total (M × N)	2,000	Cells
*g*	[0.04,0.035,0.03,0.025,0.02]	Unitless
τ	0.9	Unitless
*I*	0.3	Unitless
σ	0.24	Unitless
*T*	0.05	Unitless
ζ	Mean: 0.0, std: 0.5	Unitless

The input of the network relies on the speed vector, *v*: = (*v*_*x*_, *v*_*y*_), of the simulated agent during virtual exploration. Thus, when the agent moves, the network's activity bump shifts along the neural sheet accordingly with the agent's speed vector. Moreover, the size and the spacing of the cell's subfields are susceptible to modulation via the gain parameter *g* ∈ ℜ^+^. The network's input is thus modulated by:

(3) $$\begin{array}{c} v\to gv+\zeta \end{array}$$

In our simulations, the gain parameter *g*, defining the dorsal-to-ventral scale of each module, progressively increases the size and distance of the subfields as is found in the rodent's MEC layer 2 (Brun et al., 2008). The parameter ζ represents a uniformly distributed error perturbation on the spatial representation system.

The attractor mechanism stems from the distribution of synaptic weights of the cells organized into a 2-dimensional planar sheet. To effectively generate attractor points, the synaptic weights of a single cell with all the other cells in the network are defined by a Gaussian distribution, such that neighboring cells are connected through highly excitatory projections while distant cells connect through inhibitory projections. Thus, the synaptic weight for a given cell pair *w*_*ij*_ as a function of time is expressed as:

(4) $$w_{ij}(t)=Iexp\left(-\frac{{\left\|c_{i}-c_{j}+v(t)\right\|}_{tri}^{2}}{\sigma^{2}}\right)-T$$

where *c*_*i*_ and *c*_*j*_ express the Cartesian location of cell *i* and cell *j*, respectively in the neural sheet, and ∥*c*_*i*_ − *c*_*j*_∥ represents the Euclidean norm, or the distance between these two cells. Following Guanella et al. (2007), the intensity factor *I* defines the overall strength of the synapses, the size σ of the Gaussian modulates the synaptic distribution and the parameter *T* represents the maximum inhibitory projections of the most distal cells (see Guanella et al., 2007 for a complete description of the model and of the twisted toroidal architecture in function of ∥_*tri*_∥).

#### 2.1.2. Border cell model

**Figure 1:**
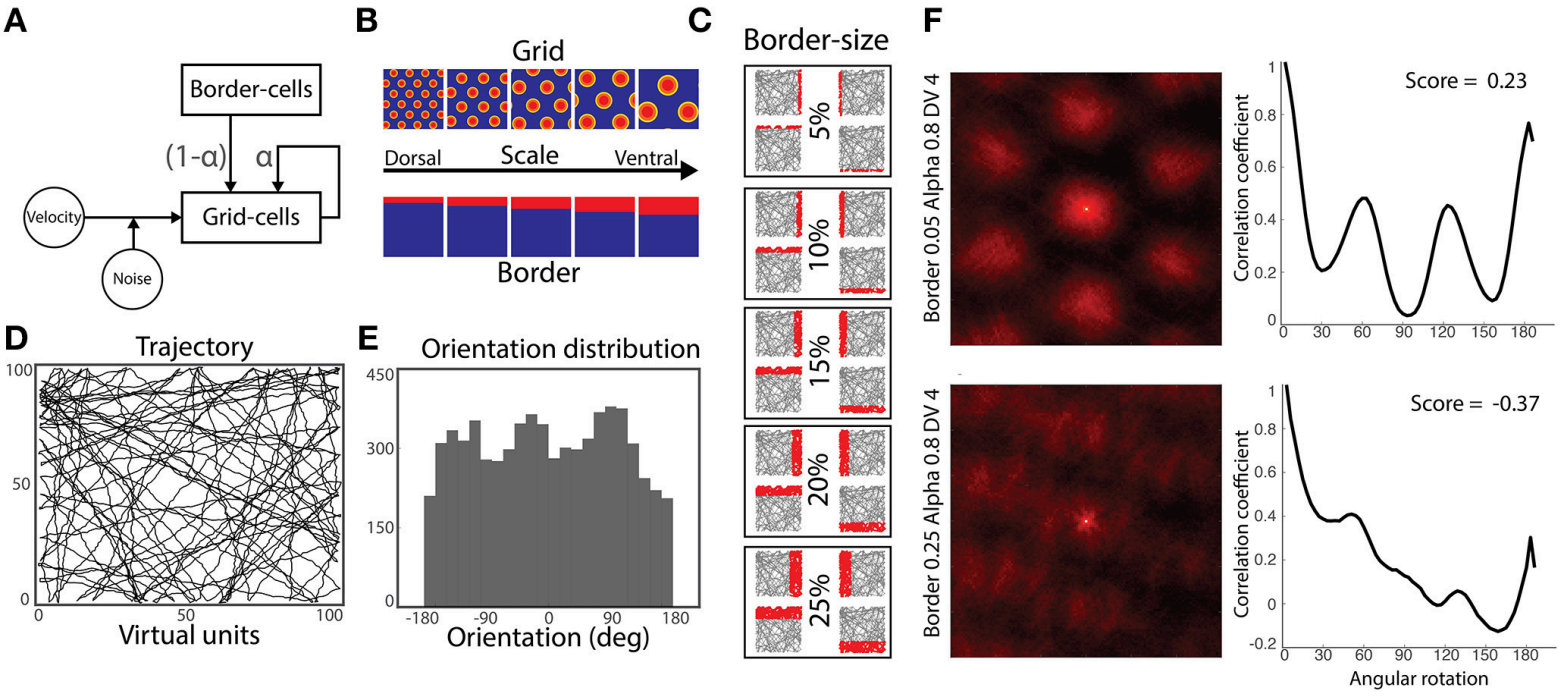
Simulation methods. **(A)** Model architecture: a population of grid cells receives noisy velocity signals disrupting their characteristic grid pattern. Simultaneously, they receive inputs from both neighbor grid cells and border cells. Gains modulating the strength of grid and border cells coupling is defined by the parameter α. **(B)** Five-by-five experimental design: grid cells and border cells express different scaling along the dorsal-to-ventral axis. **(C)** Activity of border cells in each dorsal-to-ventral scale condition. Each cell has its preferred environmental boundary (North, South, East or West). **(D)** Virtual agent's trajectories during a simulation run. **(E)** Orientation's distribution from performed trajectories of **(D)**. **(F)** Autocorrelograms of two representative grid cells' spatial activity (left) and their respective rotational correlation scores (right). Rate maps are zoomed to the central peak of the autocorrelograms denoting higher active bumps. Both cells are from the same dorsal-to-ventral scale level (fourth) and alpha condition (0.8), but different border scales (0.25 and 0.05, respectively). Oscillatory correlation from rotational measure is observed in cells with positive gridness scores (upper-right), but not in cells with negative scores (bottom-right).

Border cells' activity is algorithmically defined by a monitoring rule setting the cells as active when the agent is near their preferred environmental boundary—defined as a factor of border field amplitude. Four border cells are implemented as binary neurons and their receptive fields are tuned to specific boundaries following the cardinal directions (North, South, East and West) of the square arena. To test the effect of border fields' size on grid cells' error minimization, five simulation conditions for border cells' activity are set according to the agent's maximum distance to a wall for excitation of the border cell (5, 10, 15, 20, or 25% of the length of the arena)—see Figures 1B,C.

(5) $$A_{b}=\{\begin{array}{ll} 1, & \text{if agent position in preferred boundary}\\ 0, & \text{otherwise}. \end{array}$$

#### 2.1.3. Hebbian learning

To test the effects of border cells' activity on grid cells' error minimization, and isolate it from the learning process, the border-to-grid synaptic weights are computed before each condition and remain fixed throughout each experimental condition. These simulations are prone to noise induced velocity signals whenever error signals are present, irrespective of the learning stage.

During the learning phase, grid-to-border synaptic weights are updated accordingly by:

(6) $$\begin{array}{r} z_{ij}\left(t+1\right)=z_{ij}\left(t\right)+\eta x_{i}\left(t\right)x_{j}\left(t\right) \end{array}$$

where *z*_*ij*_ is the synaptic weight between cells *i* and *j* at time *t*, η is the learning rate, *x*_*i*_ is the presynaptic activation from border cells' activity and *x*_*j*_ is the postsynaptic grid cells' response.

### 2.2. Border to grid ratio: the alpha value

Because grid cells' populations are based on low continuous attractor dynamics in a fully connected network, implying that extensive lateral connectivity drives bumps of activity in the network, grid cells in our model receive three types of input signals: velocity-related, boundary-related from border cells, and location-related from neighboring grid cells of the same network. Given that our simulations imply multiple grid and border scale conditions, we are able to explore the effects of changing the input gains from border and grid cells on the maintenance of grid cells' hexagonal tessellation pattern.

In our simulations, each grid/border scale condition contains eleven gain modulation conditions affecting how much grid cells' activity and how much border cells' activity contribute to the final output of the grid cells.

The inherent dynamics of the model implies that adjunctive projections from neighboring grid cells at any given time step will affect a grid cell in the following time step (Equations 1–4). However, because in our model grid cells also have strengthened synapses with border cells, the effect of grid-to-grid and border-to-grid projections in modulating grid cells' activity can be manipulated through the parameter α. When integrated with the border cells' activity, *A*_*b*_, and the α parameter, the final grid cells' activity, *A*_*i*_, is given by:

(7) $$A_{i}(t+1)=\alpha {A^{\prime }}_{i}(t+1)+(1-\alpha )z_{ij}\cdot A_{b}(t+1)$$

where α reflects the strength of grid cells (α) and border cells (1 − α) in the grid cells' output signals, in the range of 0–1 with steps of 0.1. When α = 0, the output signal is modulated solely by the border cells' activity, whereas when α = 1 it would reflect pure grid-like signals. Intuitively, one could expect that a plausible α value in the rodent hippocampus would have to favor grid cells' signals but still permit sufficiently strong border cells' signals for the minimization of accumulated error. However, both the optimal grid/border ratio for error minimization and its effects for the different dorsal-to-ventral scale combinations are unknown.

#### 2.2.1. Experimental conditions

With this model, we aim to answer two questions. Firstly, would grid cells benefit from a dorsal-to-ventral scaling of border cells for minimization of path integration related error accumulation? Secondly, what are the optimal gains of inputs to grid cells for the maintenance of a positive grid score for the grid cells?

To this end, we simulate the relationship between three model parameters for a total of 275 sessions: (1) the grid scale (Figure 1B, top row); (2) the border scale (Figure 1B, bottom row) and; (3) the α value (ranging from 0.0 to 1.0) modulating the ratio of the gains of grid and border inputs (Figures 1A–C).

Across the experimental conditions defined in this study, there is a strong emphasis on the effects of border signals to maintain spatial stability in the firing activity of grid cells. Because the synaptic projections of border cells to grid cells have to be learned, one decision that needs to be made is whether the input velocity signal should contain a level of noise or not during such an associative learning process. In natural conditions, an animal is subject to noisy input both during phases of learning as well as during later phases of exploration. As we aim to quantify the impact of velocity related noise signals in the learning process, we thus added noise to the input in both conditions.

Because the grid-to-grid synaptic weights are constantly modulated by the velocity signal, allowing for the integration of the agent's navigational path to its internal representation, adding the noise signal (mean = 0.0, *SD* = 1) would simulate an accumulation of the velocity signals and consequently disrupt the grid pattern of grid cells. Thus, at every simulation step, the instantaneous directional vector, obtained from the agent's change in position from the previous step, was modulated by applying random values ranging ±0.5 virtual units in both *x* and *y* directions.

Every experimental session consists of a simulated agent randomly navigating within a square virtual arena (Figure 1D). When the agent approaches a wall, a collision avoidance mechanism is set to maintain exploration within the environmental boundaries. Thus, simulated trajectories were distributed along the global polar coordinates (Figure 1E).

For each simulation, we randomly choose 10% of the overall grid cells for the analysis. Keeping within the scope of this study, we focus only on grid cells' spatial activity. Specifically, for each cell's rate map, we calculated the gridness score, which is a measure of minimization of path integration error accumulation (Sargolini et al., 2006). Because grid cells lose their grid pattern after accumulating errors, the gridness score is a plausible measure to assess the influence of the border cells' Hebbian mechanism in resetting grid signals to their actual locations.

We first computed the cell's rate map by summing its activity within the binned (size = 10) 2-dimensional representation of the explored path and normalized it by the occupancy of each spatial bin. Next, we generated the autocorrelogram of each cell by auto-correlating its 2- dimensional rate map (Figure 1F, left column). We then applied a progressive rotation of 3° to the cell's autocorrelogram and correlated it with the original zero-rotation autocorrelogram. In the case of truly symmetrical grid cells, an oscillatory correlation signal would be generated with peaks at 60° and 120° and troughs at 30°, 90°, and 150° (Figure 1F, right column). The gridness score is then obtained by subtracting the correlation at the expected peaks and the expected troughs of the rotational correlation (Sargolini et al., 2006).

## 3. Results

**Figure 2:**
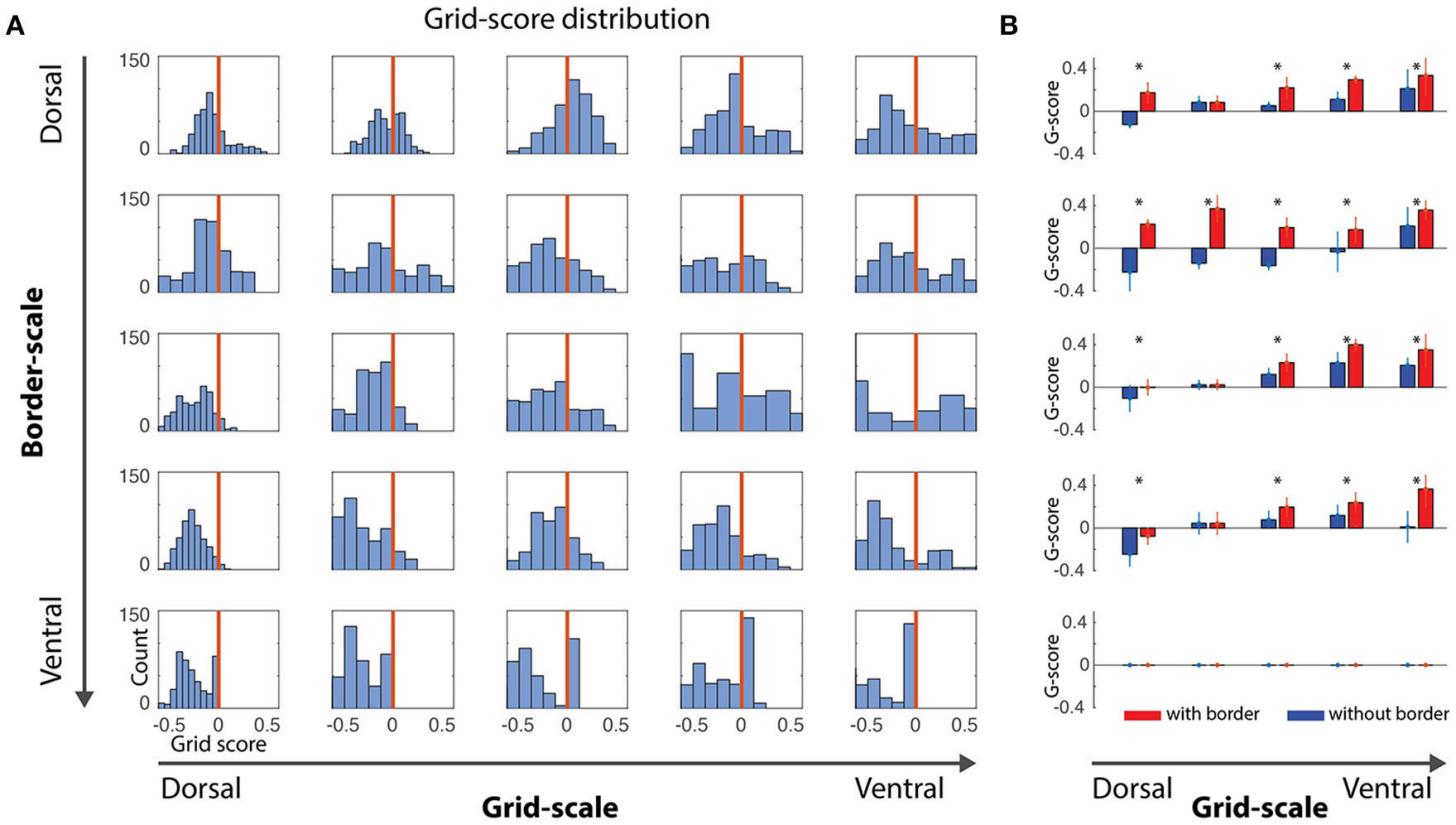
Gridness scores quantification. **(A)** Distribution of gridness scores per dorsal-to-ventral condition, independently of the α gains. Bars at the right of vertical red lines showed positive gridness and were considered grid cells. **(B)** Comparison of grid scores for conditions with- and without-border influence. With-border conditions were extracted for α values reflecting the higher grid score mean value (mean/std, ^*^where *t*-test pairwise test < 0.05), revealing that the border cell mechanism was capable of minimizing grid-cells error accumulation.

Gridness scores allow to identify whether border-to-grid Hebbian learning is effective in maintaining the hexagonal tessellation of grid cells. We first visually inspected the gridness distribution in a 2-dimensional parametric space: grid and border scale being modulated independent of the gain conditions Figure 2.

A positive gridness score implies that grid cells maintain their stereotypical pattern and, thus, that border cells help reducing the accumulated error from grid cells' path integration processes. Noise-free and noise-induced velocity signals during learning simulations revealed similar trends with respect to the border and grid scale levels, i.e., small border scales (5% and 10% of the environment width) were more effective in maintaining the typical grid cells' hexagonal pattern (Figures 2, **4**).

**Figure 3:**
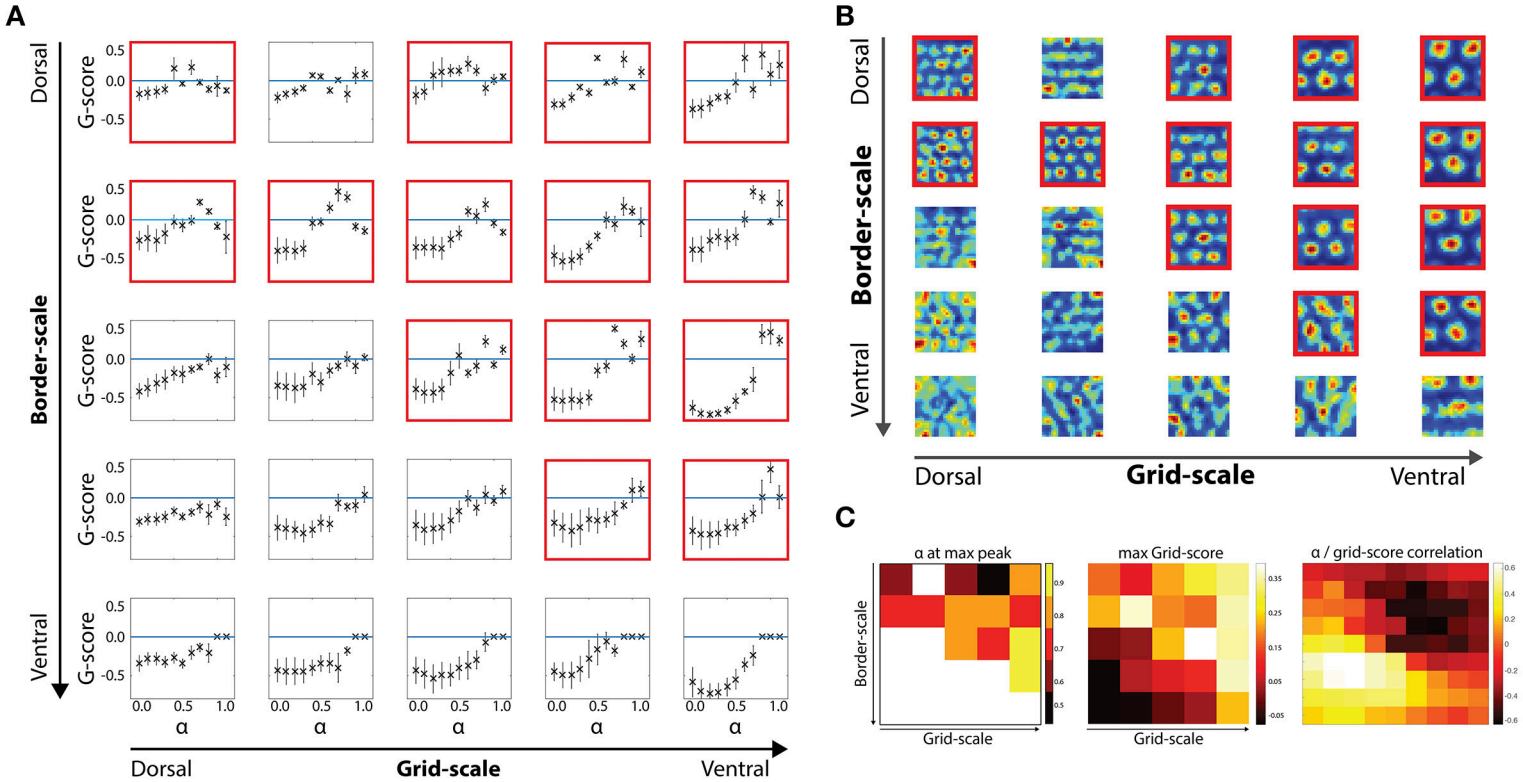
Border scale, grid scale and α modulation interactions. **(A)** Effects of the α parameter in gridness scores per dorsal-to-ventral condition with noise-induced velocity signal during the learning and testing phases. **(B)** Examples of grid cells rate maps per each scale condition. Cells were chosen based on their gridness score, so that cells with higher scores are shown. **(C)** Relationship between border and grid cells and α modulation. Modulation of border cells (alpha) as well as its effectiveness were dependent on the scale level of both border and grid cells.

As stated above, given the nature of our simulations and the challenge of quantification in this study, we focus our analyses on simulations of noise-induced velocity signals during learning. Small-scale grid cells (Figure 2A, left-most column) are unable to maintain their grid pattern when larger scaled border cells signal boundary proximities. On the other hand, as grid scale increased, the ability to maintain grid patterns progressively increased regardless of the border scale (Figure 3A), suggesting that a finer grid scale is more sensitive to border scaling for successful minimization of errors.

Another question that is addressed is whether grid cells would better benefit from across-the-board small and precise border scales or border scales that mirror the typical dorsal-to-ventral progressive increase in scale found in hippocampal head-direction, grid and place cells. Interestingly, we observe that both hypotheses could coexist within the 2-dimensional parametric space, given that the distribution of positive grid scores are higher in the upper diagonal compared to its lower band (Figures 2A, 3B,C).

We then analyzed how different values of the α gain affect cells' gridness score along the dorsal-to-ventral axis Figure 3. For low α values (range 0.0–0.4), grid cells are not able to regain positive gridness scores as border cells' activity was the predominant driver of grid cells' activity (Figure 3A).

However, at higher α values, when grid and border influences are balanced or when neighboring grid cells' activity is the predominant driver of grid cells' activity, grid cells are able to minimize path integration related errors and maintain their stereotypical hexagonal pattern (Figure 3B).

As suggested from the grid scores' distribution, at multiple grid/border scale conditions grid scores are not larger than zero, independently of the α gain values. Specifically, when the border scale is smaller than or equal to the grid scale, positive gridness scores are observed (see conditions highlighted with red frames in Figures 3A,B).

As border scale increase, cells with smaller grid scale tend to lose their tessellation, such that larger-scaled grid cells more robustly maintain their gridness when receiving input from a wider range of border scale conditions than smaller scaled grid cells.

Also, as observed earlier, large-scale border cells (covering 25% of the environmental width) are not able to contribute to a positive grid score, suggesting that border cells with low information specificity might not be useful within the hippocampal spatial representational system.

With respect to the effects of the α parameter, in our simulations it would be expected that when border cells do not contribute to the final activity of grid cells (α = 1) gridness scores would match independently of the border scale level. However, that is not found in the present results (Figure 3A). Given the parameters of our simulations, such perturbations could be due to the fact that for each border scale and α value condition, the learning between border and grid cells is modulated by the noise-induced signal during virtual navigation.

**Figure 4:**
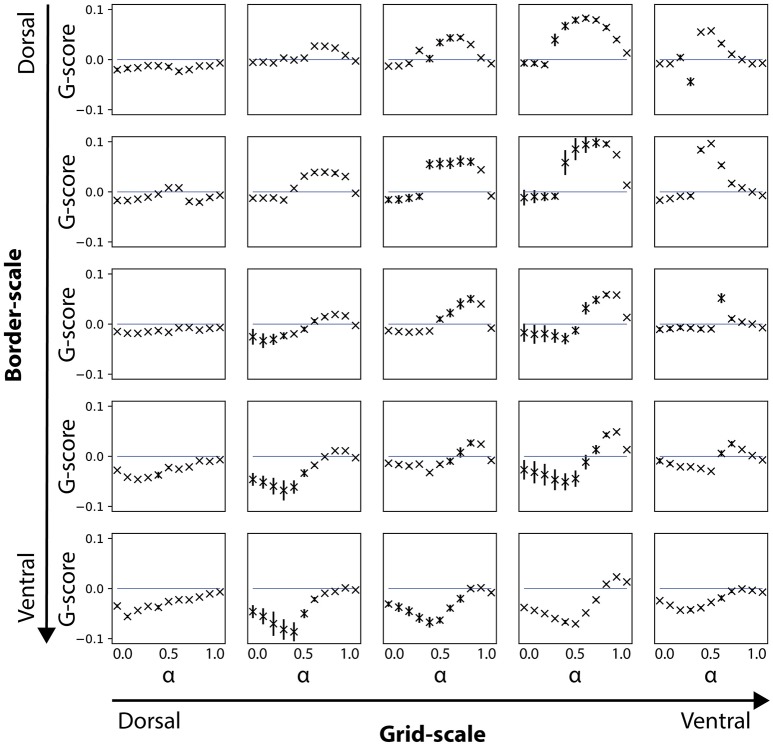
Gridness scores for noise-free velocity signal during the learning phase. Results are shown for simulations where the velocity signal during learning was noise-free, but noise-induced during the testing phase. Note that gridness scores are affected for conditions of smaller (dorsal) but not for larger (ventral) border cell scales.

To measure these effects, we run a second set of simulations where, during the learning phase, the velocity signal was error-free (Figure 4). Indeed, we verified that gridness scores at each grid cell scale level match independently of the border cells' scale when α = 1.

To quantify how much border cells contributed to grid cells' error minimization within the border/grid scale parametric space, we extracted grid scores in conditions of zero-effect from border cells and compared it with gains displaying maximal gridness score means for each border/grid scale condition (Figure 2B). Irrespective of the scaling, at every condition in which gridness was not close to zero, significant differences between with/without borders were observed (*p* < 0.01, Wilcoxon Signed-Rank test). Thus, it supports the proposed mechanism that border cells successfully contribute to the maintenance of grid patterns under noisy velocity signals input.

Again, small-scale grid cells did not produce positive gridness scores when border scales were larger. As shown before, large scale border cells disrupt the hexagonal pattern at every grid scale condition.

We next analyzed the effects of α gains modulating the strength of border and grid signal inputs in the grid cells' activity update in terms of gridness score optimality. We first extracted the gains' values in the 2-dimensional scale condition space in which at least one gain condition displayed a positive average score on the grid cells' population. As previously described, positive gridness scores were mostly obtained in the upper right quadrant of the 2-dimensional scale condition space i.e., whenever grid cells' scale is equal or higher than border cells' scale.

For the smallest boundary signals (α = [0.5:0.6]), the best gridness score was obtained at a balance between grid and border cells' input. In contrast, larger border scales led to better scores in higher α levels ([0.7:0.9]), revealing that a larger grid cell input is necessary to maintain the grid cells' hexagonal tessellation patterns (Figures 3A,C).

A dual effect was found for gridness scores in the same parametric space (Figure 3C). On the one hand, grid cells with matching border scales obtained positive scores, reflecting an optimal scale-pairing between both cell types. On the other hand, larger scaled grid cells maintained positive gridness scores independent of the border scale condition.

## 4. Conclusions and discussion

Grid cells have been extensively studied in rodent navigation. At the theoretical level, it has been argued that grid cells form a sensory-independent spatial representation and, when combined with lateral enthorinal cortex (LEC) inputs, they would allow the hippocampus proper to encode for an animal's specific location within the environment. However, recent experimental insights have shown that grid cells are modulated by environmental changes, suggesting a more interconnected representational mechanism between internally generated path integration signals and sensory processing (Krupic et al., 2015; Savelli et al., 2017).

An effective representational system needs to be stable over time and traveled distance. However, analog signals (either internally or externally generated) result in error accumulation over time that is passed to the systems they feed into. Such accumulation has been observed in rodent grid cells during navigation in enclosed environments, which leads to the question of how this accumulated error is minimized for optimal performance.

This has been addressed through the recording of spatial error accumulation in grid cells during open field navigation (Hardcastle et al., 2015), where it was observed that field traversals increase error accumulation while environmental boundaries minimize it. Thus, it raised the possibility that the representation of boundaries serves as an anchoring signal for grid cells' stabilization. A computational model allowing for the error minimization in grid cells through border cells' signals using Hebbian learning was thus proposed by the authors.

That grid cells' spatial representations exist on a range of scales along the dorsal-to-ventral axis in the MEC could affect how signals from border cells contribute to error minimization. Specifically, the ratio of the scale of the projecting border cell to that of the receptive grid cell is likely to be a factor in how well the grid cell's errors are minimized. At the physiological level, this effect remains unclear. Moreover, it also brings up the question of whether border cells exhibiting such scaling properties, may be more optimal in minimizing the accumulation of errors.

In this study, we addressed these questions by modeling the interaction between border and grid cells using low-continuous attractor dynamics. By integrating the noisy velocity signal of a simulated agent and border signals of different scales to a modeled population of grid cells, we were able to control the proportion of input into grid cells which come from border cells, velocity signals and grid cells. In our model, a single parameter α determined the contribution of border cells' activity on grid cells' response. In simulations where α = 1—that is, border cells did not contribute to the grid cells' response—positive gridness scores were still observed for the ventral (large-scale) grid cells.

Thus, we were able to quantify the effects of scaling properties (in both grid and border cells) on grid cells' error minimization. Our results suggest that equally scaled receptive fields of both grid and border cells would best minimize accumulated error when compared with larger border fields and smaller grid scale. Simply put, projections from border to grid cells at the same dorsal-to-ventral axis level would satisfy the mechanism for error minimization. Interestingly, we also observed that smaller scale border cells would just as adequately help in the maintenance of the gridness of grid cells regardless of the grid cells' scale. This suggests that border cell scaling along the dorsal-to-ventral axis is not strictly necessary for optimal error minimization, assuming that border scale is sufficiently small.

As mentioned before, Bonnevie et al. (2013) has shown that excitatory projections from the hippocampus to the MEC are required for maintaining grid cells' gridness. Those projections do not directly target grid cells populations in layers II and III but rather reach them through earlier layers of the MEC where border cells are found. In the context of error minimization, this phenomenon could therefore potentially be due to the loss of border cells' spatial tuning leading to a disruption in their role of maintaining grid cells' spatial accuracy.

An alternative solution to error minimization was proposed by Mulas et al. (2016), in which a Hebbian mechanism between grid cells and sensory cues for grid realignment resets grid cells' activity to the agent's correct location. Despite the computational benefits of such an approach, there is no physiological evidence for direct projections from the LEC sensory hub to the MEC spatial encoder. Thus, such sensory to spatial influences are likely to be filtered through hippocampal place cells before reaching grid cells and is unlikely to be the solution found in the rat hippocampus.

In conclusion, we examined how scaling could be useful to border cells in the context of error minimization in grid cells. As grid cells benefit from boundaries to minimize errors and border cells can be found one synaptic stream adjacent to grid cells, the effects of changing border scales could reveal a function of a scaling property. Our computational work suggests that both small-scale and comparable-to-grid-scaled border cells are effective in minimizing grid cells' error accumulation. High resolution, small, border cells' firing fields along the MEC dorsal-to-ventral axis might thus represent a parsimonious solution equally contributing to grid cells of multiple scales along the same axis.

## Author contributions

DS, RZ, and PV designed the study. DS implemented the simulations. DS and RZ analyzed the data. All authors contributed to the writing of the manuscript.

### Conflict of interest statement

The authors declare that the research was conducted in the absence of any commercial or financial relationships that could be construed as a potential conflict of interest.

## References

[B1] Bonnevie T. Dunn B. Fyhn M. Hafting T. Derdikman D. Kubie J. L.. (2013). Grid cells require excitatory drive from the hippocampus. Nat. Neurosci. 16, 309–317. 10.1038/nn.3311 23334581

[B2] Brown T. I. Carr V. A. LaRocque K. F. Favila S. E. Gordon A. M. Bowles B.. (2016). Prospective representation of navigational goals in the human hippocampus. Science 352, 1323–1326. 10.1126/science.aaf0784 27284194

[B3] Brun V. H. Solstad T. Kjelstrup K. B. Fyhn M. Witter M. P. Moser E. I.. (2008). Progressive increase in grid scale from dorsal to ventral medial entorhinal cortex. Hippocampus 18, 1200–1212. 10.1002/hipo.20504 19021257

[B4] Burak Y. Fiete I. R. (2009). Accurate path integration in continuous attractor network models of grid cells. PLoS Comput. Biol. 5:e1000291. 10.1371/journal.pcbi.1000291 19229307 PMC2632741

[B5] Burgess N. Maguire E. A. O'Keefe J. (2002). The human hippocampus and spatial and episodic memory. Neuron 35, 625–641. 10.1016/S0896-6273(02)00830-9 12194864

[B6] de Almeida L. Idiart M. Lisman J. E. (2009). The input–output transformation of the hippocampal granule cells: from grid cells to place fields. J. Neurosci. 29, 7504–7512. 10.1523/JNEUROSCI.6048-08.2009 19515918 PMC2747669

[B7] Domnisoru C. Kinkhabwala A. A. Tank D. W. (2013). Membrane potential dynamics of grid cells. Nature 495, 199–204. 10.1038/nature11973 23395984 PMC4099005

[B8] Giocomo L. M. Stensola T. Bonnevie T. Van Cauter T. Moser M.-B. Moser E. I. (2014). Topography of head direction cells in medial entorhinal cortex. Curr. Biol. 24, 252–262. 10.1016/j.cub.2013.12.002 24440398

[B9] Giocomo L. M. Zilli E. A. Fransén E. Hasselmo M. E. (2007). Temporal frequency of subthreshold oscillations scales with entorhinal grid cell field spacing. Science 315, 1719–1722. 10.1126/science.1139207 17379810 PMC2950607

[B10] Guanella A. Kiper D. Verschure P. (2007). A model of grid cells based on a twisted torus topology. Int. J. Neural Syst. 17, 231–240. 10.1142/S0129065707001093 17696288

[B11] Hafting T. Fyhn M. Molden S. Moser M.-B. Moser E. I. (2005). Microstructure of a spatial map in the entorhinal cortex. Nature 436, 801–806. 10.1038/nature03721 15965463

[B12] Hardcastle K. Ganguli S. Giocomo L. M. (2015). Environmental boundaries as an error correction mechanism for grid cells. Neuron 86, 827–839. 10.1016/j.neuron.2015.03.039 25892299

[B13] Julier S. J. Uhlmann J. K. (1997). New extension of the Kalman filter to nonlinear systems, in AeroSense'97, International Society for Optics and Photonics (Orlando, FL), 182–193.

[B14] Kam M. Zhu X. Kalata P. (1997). Sensor fusion for mobile robot navigation. Proc. IEEE 85, 108–119. 10.1109/JPROC.1997.554212

[B15] Kjelstrup K. B. Solstad T. Brun V. H. Hafting T. Leutgeb S. Witter M. P.. (2008). Finite scale of spatial representation in the hippocampus. Science 321, 140–143. 10.1126/science.1157086 18599792

[B16] Kropff E. Carmichael J. E. Moser M.-B. Moser E. I. (2015). Speed cells in the medial entorhinal cortex. Nature 523, 419–424. 10.1038/nature14622 26176924

[B17] Krupic J. Bauza M. Burton S. Barry C. OKeefe J. (2015). Grid cell symmetry is shaped by environmental geometry. Nature 518, 232–235. 10.1038/nature14153 25673417 PMC4576734

[B18] Lynen S. Achtelik M. W. Weiss S. Chli M. Siegwart R. (2013). A robust and modular multi-sensor fusion approach applied to mav navigation, in Intelligent Robots and Systems (IROS), 2013 IEEE/RSJ International Conference on (Tokyo: IEEE), 3923–3929.

[B19] Maffei G. Santos-Pata D. Marcos E. Sánchez-Fibla M. Verschure P. F. (2015). An embodied biologically constrained model of foraging: from classical and operant conditioning to adaptive real-world behavior in dac-x. Neural Netw. 72, 88–108. 10.1016/j.neunet.2015.10.004 26585942

[B20] Markus E. J. Barnes C. A. McNaughton B. L. Gladden V. L. Skaggs W. E. (1994). Spatial information content and reliability of hippocampal ca1 neurons: effects of visual input. Hippocampus 4, 410–421. 10.1002/hipo.450040404 7874233

[B21] Moser E. I. Kropff E. Moser M.-B. (2008). Place cells, grid cells, and the brain's spatial representation system. Annu. Rev. Neurosci. 31, 69–89. 10.1146/annurev.neuro.31.061307.090723 18284371

[B22] Mulas M. Waniek N. Conradt J. (2016). Hebbian plasticity realigns grid cell activity with external sensory cues in continuous attractor models. Front. Comput. Neurosci. 10:13. 10.3389/fncom.2016.00013 26924979 PMC4756165

[B23] Naber P. A. Lopes da Silva F. H. Witter M. P. (2001). Reciprocal connections between the entorhinal cortex and hippocampal fields ca1 and the subiculum are in register with the projections from ca1 to the subiculum. Hippocampus 11, 99–104. 10.1002/hipo.1028 11345131

[B24] O'keefe J. Nadel L. (1978). The Hippocampus as a Cognitive Map. Oxford: Clarendon Press.

[B25] Pata D. S. Escuredo A. Lallée S. Verschure P. F. (2014). Hippocampal based model reveals the distinct roles of dentate gyrus and ca3 during robotic spatial navigation, in Conference on Biomimetic and Biohybrid Systems (Milan: Springer), 273–283.

[B26] Sargolini F. Fyhn M. Hafting T. McNaughton B. L. Witter M. P. Moser M.-B.. (2006). Conjunctive representation of position, direction, and velocity in entorhinal cortex. Science 312, 758–762. 10.1126/science.1125572 16675704

[B27] Savelli F. Luck J. Knierim J. J. (2017). Framing of grid cells within and beyond navigation boundaries. eLife 6:e21354. 10.7554/eLife.21354 28084992 PMC5271608

[B28] Solstad T. Boccara C. N. Kropff E. Moser M.-B. Moser E. I. (2008). Representation of geometric borders in the entorhinal cortex. Science 322, 1865–1868. 10.1126/science.1166466 19095945

[B29] Squire L. R. (1992). Memory and the hippocampus: a synthesis from findings with rats, monkeys, and humans. Psychol. Rev. 99:195. 10.1037/0033-295X.99.2.195 1594723

[B30] Yoon K. Buice M. A. Barry C. Hayman R. Burgess N. Fiete I. R. (2013). Specific evidence of low-dimensional continuous attractor dynamics in grid cells. Nat. Neurosci. 16, 1077–1084. 10.1038/nn.3450 23852111 PMC3797513

